# Clinicopathological features of invasive micropapillary carcinoma of the breast

**DOI:** 10.3892/ol.2014.2806

**Published:** 2014-12-17

**Authors:** ZHAO-QING CUI, JI-HUAN FENG, YI-JUN ZHAO

**Affiliations:** Department of Breast and Thyroid Surgery, Liaocheng People’s Hospital of Shandong Province, Liaocheng, Shandong 252000, P.R. China

**Keywords:** breast carcinoma, invasive micropapillary carcinoma, immunohistochemistry

## Abstract

The aim of the present study was to evaluate the clinical and immunohistopathological findings of invasive micropapillary carcinoma (IMPC) of the breast. In total, 25 patients were included in the present study, all of whom were diagnosed with IMPC. The mammography and ultrasound scanning (US) findings were analysed retrospectively according to the American College of Radiology Breast Imaging Reporting and Data System lexicon. Surgical specimens obtained from the patients were microscopically reviewed in consensus by two pathologists with a specialisation in breast pathology. All the patients presented with palpable lumps in the breast, a high-density irregular mass associated with microcalcifications revealed by mammography and an irregular hypoechoic mass with a spiculated margin revealed by US. Axillary lymph node metastases were identified in 80% of the patients. Immunohistochemical studies revealed the lesions to be highly positive for the oestrogen receptor (ER) and c-erbB-2 (88% and 84%, respectively). Although no significant imaging characteristics were found to distinguish IMPC from typical invasive ductal carcinoma, IMPC resulted in nodal metastases and was highly positive for ER and c-erbB-2. This clinical significance indicates the significance of this entity being recognised by pathologists and surgeons.

## Introduction

Invasive micropapillary carcinoma (IMPC) of the breast is a morphologically distinct and aggressive variant of invasive ductal carcinoma (IDC), accounting for <2% of all invasive breast cancer cases (1). Morphologically, IMPC exhibits a peculiar architecture characterised by pseudopapillary structures that are composed of cell clusters with inverted polarity floating in empty spaces and lined by delicate strands of fibrous stroma (2). IMPC was first described in the literature by Petersen in 1993 (3). In the 2003 World Health Organisation (WHO) classification of breast tumours (4), IMPC was listed as a subtype of invasive carcinoma (1). However, in the literature, no consensus has been reached regarding the amount of IMPC tissue in the breast carcinoma required to make a diagnosis and determine the type of IMPC (5). IMPC is associated with a high incidence of axillary lymph node metastases and local recurrence and poor clinical outcome (6). These clinical characteristics indicate the significance of IMPC being recognised by surgeons and pathologists. Therefore, the clinical and immunohistochemical characteristics of IMPC were retrospectively examined.

## Materials and methods

### Patient selection

In total, the records of 25 patients diagnosed with IMPC were retrieved from the histopathological medical records from the Department of Pathology of Liaocheng People’s Hospital of Shangdong Province (Liaocheng, China). These patients presented over a six-year period, between July 2005 and July 2011. The present study was conducted in accordance with the Declaration of Helsinki and with approval from the Ethics Committee of Liaocheng People’s Hospital. Written informed consent was obtained from all participants.

### Immunohistochemical staining

Haematoxylin and eosin were used to stain 10% of formalin-fixed, paraffin-embedded 4-μm tissue sections. Immunohistochemical studies on oestrogen receptor (ER), progesterone receptor (PR), c-erbB-2 and epithelial membrane antigen (EMA) were conducted. The slides were incubated overnight at 55°C to enhance the adhesion of the sections to the slides. Deparaffinisation in xylene and graded alcohol followed. Prediluted monoclonal rabbit anti-human ER (1:240), monoclonal rabbit anti-human PR (1:240), polyclonal rabbit anti-human c-erbB-2 (1:400) and monoclonal EMA (1:400) antibodies were obtained from the Maixin Biotechnology Development Co., Ltd. (Fuzhou, Fujian, China). The primary antibodies were applied for 60 min at room temperature, then the slides were washed with phosphate-buffered saline (PBS) three times for 5 min each. Next, the secondary mouse anti-rabbit monoclonal secondary antibodies (RMA-0501, RMA-0502, RMA-0156 and KIT-0011; Maixin Biotechnology Development Co., Ltd.) was added and the slides were washed three times with PBS for 3 min each. 3,3′-diaminobenzidine (Maixin Biotechnology Development Co., Ltd.) was then added and the slides were visualized under a microscope (Eclipse 80i; Nikon Corporation, Tokyo, Japan).

### Immunohistochemical evaluation

Using light microscopy, stained tissue sections were reviewed by two pathologists blind to the diagnosis. All unclear cases were discussed with an additional pathologist. Morphologically, IMPC exhibited a peculiar architecture characterised by pseudopapillary structures that were composed of cell clusters with inverted polarity and floating in empty spaces and lined by delicate strands of fibrous stroma. The cases were fully characterised on the basis of morphological features described in the original articles on IMPC by Tavassoli and Devilee (1) and Fisher *et al* (7). Histological grading was performed using the modified Bloom-Richardson grading system. Immunohistochemical studies were carried out to determine the characteristic pattern of EMA expression in IMPC to support the diagnosis. ER and PR receptor status tests were performed routinely, and the HER-2/neu status was determined by immunohistochemistry (IHC), with or without fluorescence *in situ* hybridisation (FISH), when requested by the clinician. The evaluation of the ER, PR and HER-2 statuses was conducted in accordance with the US guidelines (8).

## Results

### Clinical data

All the 25 patients were female. The mean age of the patients was 52.3 years and the age range was 34–79 years. The initial manifestation of carcinoma was a palpable mass in 21 patients (84%), a palpable mass with nipple discharge in two patients (8%) and a screening mammographic abnormality in two patients (8%). The breast cancer lesion was located in the left breast in 14 patients (56%) and in the right breast in 11 patients (44%). All the patients underwent mammography. Masses were noted in 23 patients (92%), microcalcifications were observed in 15 patients (60%) and microcalcifications without mass or asymmetry were observed in two patients (8%). The masses were found to be irregular in shape with a high density and a non-circumscribed margin, with the latter two patients exhibiting fine pleomorphic microcalcifications. Ultrasound scanning (US) was performed in all 25 cases. The masses exhibited an irregular shape with a speculated margin in 23 patients (92%), a hypoechoic pattern in 21 patients (84%) and posterior acoustic shadowing in 17 patients (68%). On the basis of the Breast Imaging-Reporting and Data System final assessment (9), 23 patients (92%) were classified as category 5 and two cases were classified as category 4 (8%). The axillary lymph nodes in all 25 patients were also examined, with 13 patients (52%) suspected to possess lymph node metastasis. All 13 patients were confirmed to possess lymph node metastasis upon pathological examination.

### Pathological results

Seven patients that were negative for metastasis on the US of the axillary lymph node were found to possess metastatic lymph nodes upon pathological examination. Fine-needle aspiration cytology of the breast was sought in eight of the cases and six of these patients were diagnosed as carcinoma. Core-needle biopsy was also performed in 18 patients, 16 of which were diagnosed with carcinoma and five of these 16 cases were diagnosed with IMPC. In total, 25 patients underwent modified radical mastectomy and two patients underwent a lumpectomy. Of the 25 surgical specimens, lymphovascular invasion was found in 11 (44%) patients, and axillary lymph node metastases were identified in 20 (80%) cases. The mean number of metastatic axillary lymph nodes was 5.7 (range, 3–21).

### Immunohistochemical evaluation

**Figure 1:**
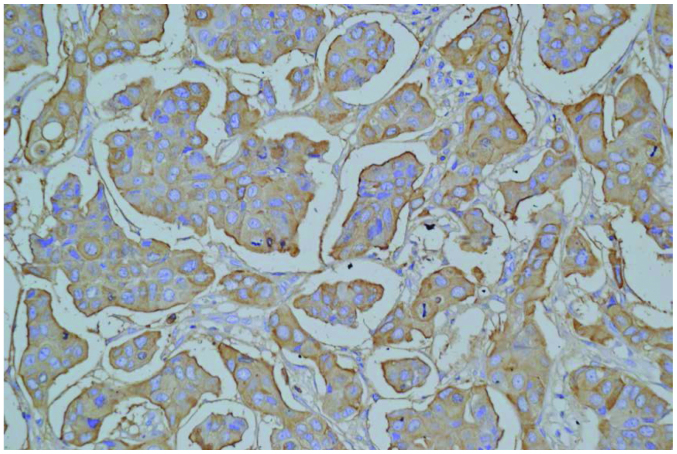
Invasive micropapillary carcinoma of the breast exhibiting a characteristic pattern of immunohistochemical epithelial membrane antigen expression; staining in the peripheral cells of the tumour clusters and the borders of the stromal spaces. Immunohistochemical staining; magnification, ×400.

Immunohistochemical analyses were carried out in all 25 cases. The findings revealed the expression of ER in 88% of the cases (22/25) and PR in 64% of the cases (16/25). In terms of c-erbB-2, an IHC score of 0 was demonstrated in one case (4%), 1+ in two cases (8%), 2+ in six cases (24%) and 3+ in 16 cases (64%). Five out of the six cases with a score of 2+ were revealed by FISH to possess gene amplification. Therefore, overexpression of the c-erbB-2 protein was observed in 84% of the cases (21/25). Immunohistochemical studies were carried out in all cases to determine the characteristic pattern of EMA expression to support the diagnosis of IMPC (Fig. 1).

### Follow-up

Follow-up information was available for all 25 patients. The mean follow-up period was 36.5 months (range, 1–55 months). Recurrence was noted in three patients, with one recurrence in the contralateral breast tissues and two in the liver and lung. Of the latter two patients, one succumbed to the disease after seven months and the other succumbed after 29 months.

## Discussion

**Figure 2:**
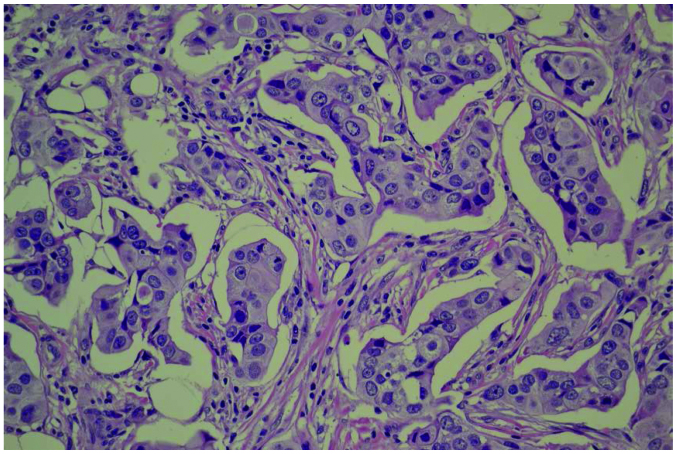
Histological features of invasive micropapillary carcinoma are characterised by pseudopapillary structures floating in empty spaces that are lined by delicate strands of fibrous stroma. Haematoxylin and eosin staining; magnification, ×400.

IMPC is an uncommon, clinically aggressive variant of IDC (10) that accounts for 0.7–3% of all cases of breast cancer. Morphologically, IMPC exhibits a peculiar architecture characterised by pseudopapillary structures that consist of cell clusters with inverted polarity floating in empty spaces and lined by delicate fibrous stroma strands (Fig. 2) (2). The 2003 WHO classification of breast tumours listed IMPC as a subtype of invasive carcinoma. However, there was no percentage of the IMPC component within the tumour that was proposed as a criterion for diagnosis (1). Certain experts hypothesise that the size of the IMPC structure should be >5 mm (11,12). Other studies support that the percentage of the IMPC should be >75%. The literature has not reached a consensus regarding the amount of IMPC necessary for a diagnosis and determining the type of IMPC (13). Despite only a few studies on IMPC imaging findings being conducted, it has been previously reported that mammography may reveal IMPC as an irregular, speculated or indistinct high-density mass and that US may reveal IMPC as an irregular, indistinct or hypoechoic mass (14). In the present study, masses were also the most frequent mammographic finding. The masses were characterised by an irregular shape and speculated margin in 23 patients (92%) and a hypoechoic pattern in 21 patients (84%). The present results agree with those of a previous study, in which microcalcifications were reported in 43–68% of cases (15). In the present study, microcalcifications were identified in 60% (15/25) of the patients. Tumours with any amount of this unique histological pattern, regardless of the extent of the micropapillary component, exhibit a more aggressive clinical behaviour and possess a poorer prognosis, with a high degree of lymph node involvement (16). An invasive micropapillary pattern has been described in tumours in several organs, including the ovaries, salivary glands, colon, bladder and lungs. A clinical finding is always associated with a poor prognosis and a high incidence of lymph node metastasis (17). The mechanisms that result in the development of metastatses are variable and require several steps, including loss of contact with neighbouring cells, penetration of vessel walls, adhesion at the novel localisation and angiogenesis. Although numerous studies have attempted to explain the peculiar features of these tumours, the basis of their biological behaviour remains unclear and additional efforts have to be made to improve the understanding of this rare variant of carcinoma. Previous studies have identified that heat shock protein 27 and L1 cell adhesion molecules play an important role in the formation of the specific pathological morphology of IMPC and in the high rate of lymph node metastasis (18,19). Cluster of differentiation (CD)24 has gained much attention due to its important role in the development of cancer metastases and as a marker of malignancy in several tumour types (20). One study has demonstrated that IMPC may represent a distinct entity of breast carcinoma that exhibits a high expression of CD24 compared with IDC. This finding may explain the high lymph-vascular invasion propensity and high metastatic capability of these tumours and may be a useful tool as a future target of therapy (21). In the present study, 80% (20/25) of the patients were confirmed to possess lymph node metastasis upon pathological examination. Lymphovascular invasion was found in 11 patients (44%).

Although an ER- and PR-positive status is generally associated with an improved differentiation of tumours and an improved outcome (15), IMPC appears to be an exception. This tumour type is characterised by higher rates of ER and PR expression compared with other types (22). Zekioglu *et al* (16) reported that 68 and 61% of lesions were positive for ER and PR, respectively, in IMPC. These results are higher compared with those of common breast cancers. The reported prevalence of c-erbB-2 was slightly higher than that of IDCs. Walsh and Bleiweiss (23) reported high percentages of ER and PR positivity (90 and 70%, respectively) and nearly double the expected percentage of c-erbB-2 positivity (60%). The present study revealed ER expression in 88% of the cases (22/25) and PR expression in 64% of the cases (16/25). In terms of c-erbB-2, an IHC score of 0 was revealed in one case (4%), 1+ in two cases (8%), 2+ in six cases (24%) and 3+ in 16 cases (64%). Five out of the six cases that scored 2+ were revealed to possess gene amplification by FISH. Overexpression of the c-erbB-2 protein was observed in 84% of the cases (21/25).

The preferred treatment for IMPC is mastectomy and axillary clearance, with the addition of adjuvant chemotherapy for the treatment of patients with lymph node metastasis or a tumour size of >1 cm (24). A study that reported 72 cases of IMPC (11) revealed that IMPC is more frequently associated with lymphovascular invasion, extracapsular extension from the lymph node, a high nuclear grade and an increased degree of locoregional recurrence, particularly in the axilla and supraclavicular regions. Therefore, axillary and supraclavicular radiation therapy should be considered as a treatment for IMPC patients that possess axillary node metastasis.

In summary, the imaging findings of IMPC through mammography and US strongly suggested malignancy. There was no identification of features that distinguish IMPC from typical IDC, but the presence of frequent nodal metastases and a high positive result subsequent to ER, PR and c-erbB-2 testing was identified in IMPC. These characteristics could aid in the treatment and evaluation of the prognosis of patients with IMPC.
